# Remote Rating of Atopic Dermatitis Severity Using Photo-Based Assessments: Proof-of-Concept and Reliability Evaluation

**DOI:** 10.2196/24766

**Published:** 2021-05-25

**Authors:** Zarqa Ali, Kristina Melbardis Joergensen, Anders Daniel Andersen, Andrei Chiriac, Theis Bjerre-Christensen, Ionela Manole, Ana-Maria Dutei, Irina Deaconescu, Alina Suru, Adina Serban, Ari Pall Isberg, Priyanka Dahiya, Simon Francis Thomsen, John Robert Zibert

**Affiliations:** 1 Department of Dermato-Venereology and Wound Healing Centre Bispebjerg Hospital Copenhagen Denmark; 2 Studies&Me Copenhagen Denmark; 3 Omhu A/S Copenhagen Denmark; 4 Department of Biomedical Sciences University of Copenhagen Copenhagen Denmark

**Keywords:** atopic dermatitis, eczema, remote assessment, photo, photographs, EASI, SCORAD, severity, assessment, agreement

## Abstract

**Background:**

Digital imaging of dermatological patients is a novel approach to remote assessment and has recently become more relevant since telehealth and remote decentralized clinical trials are gaining ground.

**Objective:**

We aimed to investigate whether photographs taken by a smartphone are of adequate quality to allow severity assessments to be made and to explore the usefulness of an established atopic dermatitis severity assessment instrument on photograph evaluation.

**Methods:**

During scheduled visits in a previously published study, the investigating doctor evaluated the severity of atopic dermatitis using the Scoring AD (SCORAD) index and took photographs of the most representative lesions (target lesions) with both a smartphone and a digital single-lens reflex camera (DSLR). The photographs were then assessed by 5 dermatologists using the intensity items of the SCORAD (iSCORAD), which consists of erythema, oedema/papulation, excoriations, lichenification, oozing/crusts, and dryness (scale 0-3, maximum score 18). The mean iSCORAD of the photographs was calculated and compared with in-person assessments using Pearson correlation and Bland-Altman plots. Intraclass correlation coefficients were used for interrater reliability.

**Results:**

A total of 942 photographs from 95 patients were assessed. The iSCORAD based on smartphone photographs correlated strongly with the evaluations performed in person (iSCORAD: r=0.78, *P*<.001; objective SCORAD: r=0.81, *P*<.001; and total SCORAD: r=0.78, *P*<.001). For iSCORAD specifically, a Bland-Altman plot showed a difference in mean score of 1.31 for in-person and remote iSCORAD. In addition, the interrater agreement between the 5 rating dermatologists was 0.93 (95% CI 0.911-0.939). A total of 170 lesions were photographed, and the difference in mean scores was 1.32, 1.13, and 1.43 between in-person and remote evaluations based on photographs taken by a DSLR camera, a smartphone without flash, and a smartphone with flash, respectively.

**Conclusions:**

In terms of quality, remote atopic dermatitis severity assessments based on photographs are comparable to in-person assessments, and smartphone photos can be used to assess atopic dermatitis severity to a similar degree as photographs from a DSLR camera. Further, the variation in how the dermatologists in this study rated the iSCORAD based on the photographs was very low.

## Introduction 

Digital imaging of dermatological patients is a novel approach to remote assessment and has recently become more relevant since telehealth and remote clinical trials are gaining ground.

Clinical trials are a cornerstone of drug development and provide scientific evidence on safety and efficacy of a new pharmaceutical drug. However, traditional clinical trials take a long time to complete and are expensive and inefficient in terms of high dropout rates [1]. Fully decentralized virtual clinical trials (VCTs) that incorporate remote outcome assessments may accelerate clinical trials, increase adherence, reduce dropout rates, and bring new treatments to the market faster [2]. Teledermatology has grown over the last two decades, and the visual nature of dermatology makes it ideal for the practice of telemedicine. Teledermatology is cost-effective [3], effective in managing dermatologic diseases [4], has better diagnostic accuracy [5], and is satisfying for both patients and providers [6].

The foundation for both VCTs in dermatology and teledermatology is remote assessment, including digital assessment of photographs of skin conditions. However, little is known about remote assessment of many dermatological diseases including atopic dermatitis (AD).

Several assessment tools have been developed to grade AD severity in the clinic. Although many of these tools have been validated when used in in-person settings, it is unknown to what extent they can be applied to assess photographs remotely. To our knowledge, only one study by Hughes et al [7] has investigated the concordance between assessment of AD in person compared to a standardized set of full-body digital photographs captured by a clinical research coordinator. They reported an excellent agreement between in-person assessment and remote assessment of photographs with respect to body surface area, Eczema Area and Severity Index, and Scoring AD (SCORAD) scales. However, to better accommodate the promises of VCT in which most of the study tasks are conveniently performed on participants' smartphones from the comfort of their own home, the number of photographs required from participants in dermatological trials should be minimized.

The primary objective of this study was to investigate whether photographs taken by a smartphone are of adequate quality to allow severity assessment to be made. The secondary purpose was to determine whether SCORAD can be applied to the evaluation of photographs.

## Methods

### Data Collection

The data used in the present study were from a previously published study (Atopisk Dermatit Eksem Studie [ADES]) [5]. The study was originally designed to investigate adherence to treatment using a memory button with an associated smartphone app (Klikkit, The HabLab ApS) among patients with AD. Although not originally designed for this purpose, data from in-person severity assessments together with digital photographs of lesions taken by the doctor have been evaluated for further analysis. A medical doctor trained in AD assessment by a certified dermatologist evaluated AD severity using SCORAD [8,9] during 2 scheduled in-person visits in the clinic. The doctor took digital photographs of AD lesions using both a smartphone (with and without flash) and a digital single-lens reflex camera (DSLR). These photographs were used for severity assessment by 5 blinded dermatologists for the purpose of this analysis.

The number of target lesions from each patient was selected based on the overall number of active lesions present as determined by the investigator during the first ADES visit. If a patient had 2 active lesions, both of these were photographed. In cases of >2 active lesions, the investigator made an overall judgment of which ones to photograph based on lesion size and the presence of the following clinical signs: (1) excoriation, (2) oozing, (3) erythema, (4) lichenification, (5) dryness, and (6) swelling.

### Photograph-Based Severity Assessment

Elements from SCORAD were used to determine the severity of AD from all of the individual photographs. SCORAD is a clinical scoring tool composed of both a subjective (itch and sleep quality) and an objective part (objSCORAD) [10]. ObjSCORAD consists of evaluations of both disease intensity and extent. The intensity part of the SCORAD (iSCORAD) is based on the rating of the following 6 items: erythema, oedema/papulation, excoriations, lichenification, oozing/crusts, and dryness. These items were used to assess all the photographs to obtain a remote iSCORAD. Each item can be graded on a scale of 0 to 3, and the overall intensity score can therefore vary from 0 to 18. Each photograph was presented independently and in a random order to 5 blinded dermatologists on an iPad, on which the dermatologists would rate each of the 6 items on a scale from 0 to 3 and thereby assign a remote iSCORAD. In cases when one item could not be rated from the photograph, the dermatologist would choose “not applicable” for that specific item and the entire photo was consequently discarded.

### Statistical Analysis

To calculate one single iSCORAD per patient, the mean of all available photographs for a patient was calculated. This iSCORAD was compared with the clinical assessment performed in person. To investigate the concordance between in-person and photo-based severity assessments, Pearson correlation and Bland Altman plots were performed. To examine the relationship between photo-based assessments and the total severity scoring performed in person, Pearson correlations were used to compare photo-based iSCORAD vs in-person iSCORAD, objSCORAD, and total SCORAD, respectively. Bland-Altman plots were constructed to calculate the average bias and limits of agreement between the methods. To investigate the interrater reliability of the photo-based severity ratings, the intraclass correlation coefficient (ICC) was used. This was performed using the *icc* function from the *irr* package in R (The R Foundation) [11] and included 95% CI values. For interrater reliability, the ICC estimates were based on two-way random-effects models, absolute agreement, and average measure. An ICC >0.90, 0.75-0.90, 0.50-0.75, and <0.50 indicate an excellent, good, moderate, and poor agreement, respectively [12]. Statistical analyses were performed using the computing environment R (R Core Team) and RStudio (RStudio, PBC). 

## Results 

Of the 95 participants who were assessed by the investigator using the SCORAD in clinic, 50 (52%) were categorized as having mild AD, 36 (38%) as having moderate AD, and 10 (10%) as having severe AD. From these, a total of 942 photos were evaluated by all 5 dermatologists. The median number of photographed lesions per patient was 3 (range 2-4).

### In-Person Assessment vs Remote Assessment 

**Figure 1:**
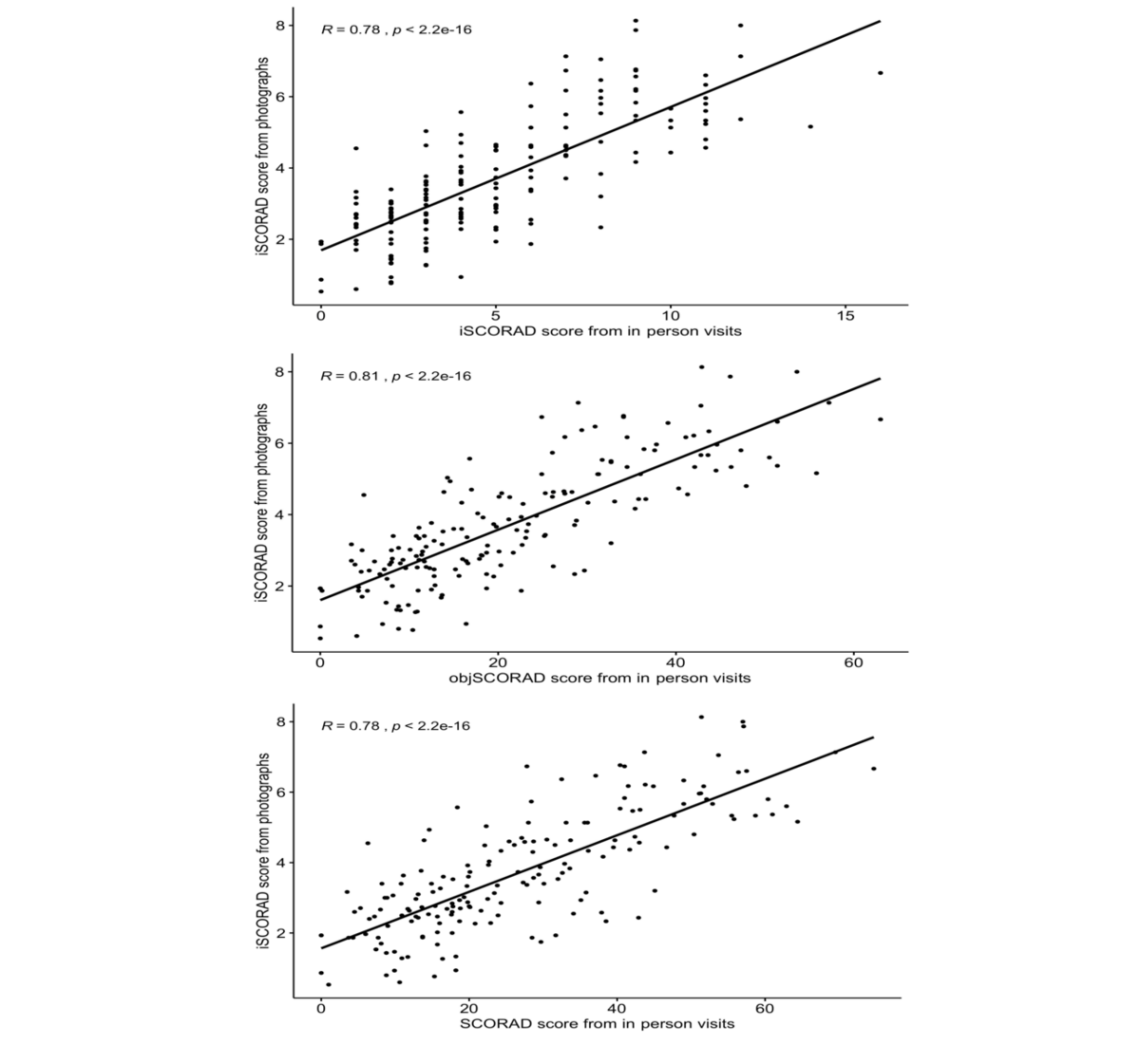
Correlation between the intensity items of Scoring AD (iSCORAD; 0-18) assessed from photos and in-person assessments based on (A) the intensity items of SCORAD, (B) the objective SCORAD (objSCORAD), and (C) total SCORAD.

The smartphone-based iSCORAD correlated strongly with the iSCORAD rated in person (r=0.78, *P*<.001). In addition, the remote iSCORAD correlated strongly with the objSCORAD (r=0.81, *P*<.001), and total SCORAD (r=0.78, *P*<.001) obtained in person (Figure 1).

**Figure 2:**
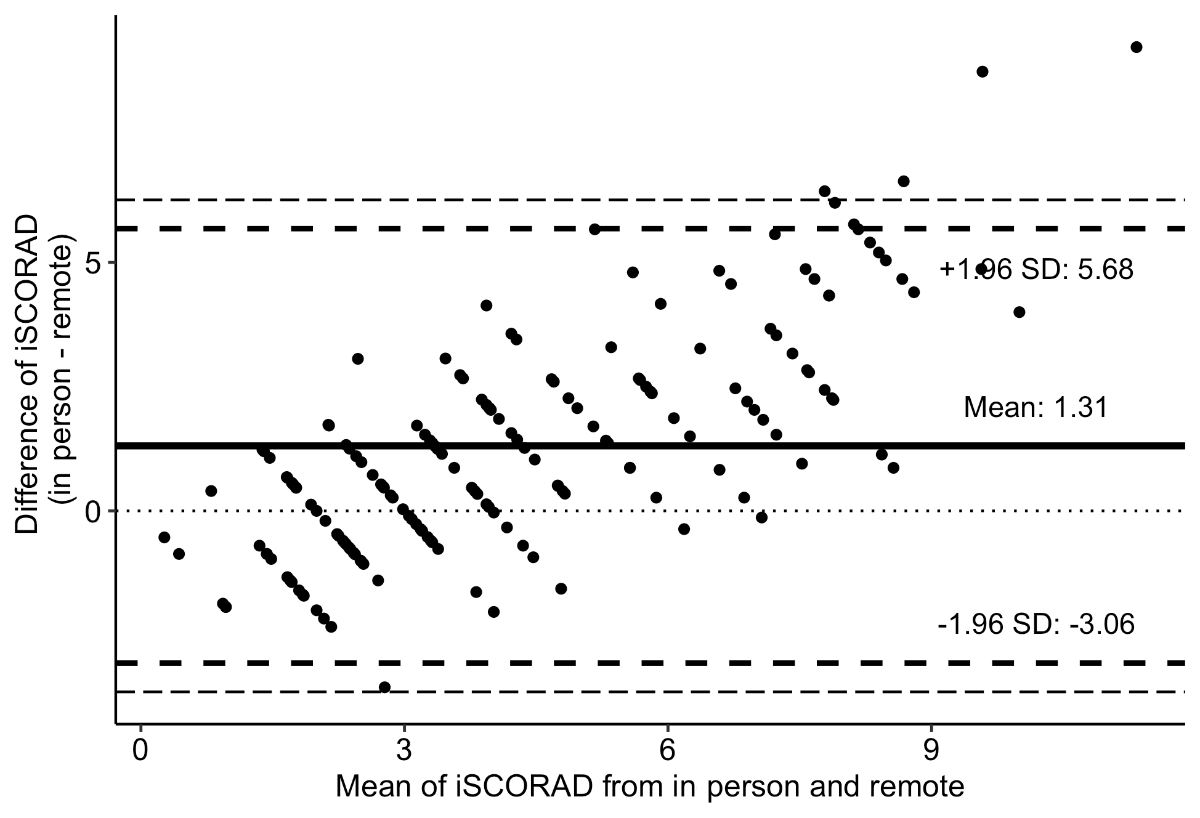
A Bland-Altman plot analyzing the difference between the intensity items of Scoring AD (iSCORAD) assessed in person and from photographs taken via smartphone. The solid line represents the mean difference, the broken line the 1.96 SD, and the dotted line represents zero.

The difference in mean scores for the Bland-Altman plot for the comparison between in-person and remote iSCORAD was 1.31 (Figure 2).

The interrater agreement between the 5 dermatologists assessing the photographs remotely was 0.93 (95% CI 0.911-0.939) (Table 1).

**Table 1:** Interrater agreement for the remote assessments done by 5 dermatologists for the intensity items of Scoring AD (iSCORAD) for different camera types.

Photographs	ICC^a^ (95% CI)
All photographs	0.926 (0.911-0.939)
DSLR^b^	0.932 (0.913-0.947)
Smartphone without flash	0.919 (0.894-0.938)
Smartphone with flash	0.926 (0.908-0.941)

^a^ICC: intraclass correlation coefficient.

^b^DSLR: digital single-lens reflex.

### Comparison of the Different Camera Types

In total, 170 lesions were photographed with all 3 camera types (ie, DSLR camera, smartphone without flash, and smartphone with flash). The difference in mean scores was 1.32 (95% CI –3.08 to 5.71), 1.13 (95% CI –3.27 to 5.53), and 1.43 (95% CI –3.05 to 5.92) between in-person evaluation and remote evaluation based on photographs taken by a DSLR camera, a smartphone without flash, and a smartphone with flash, respectively.

**Figure 3:**
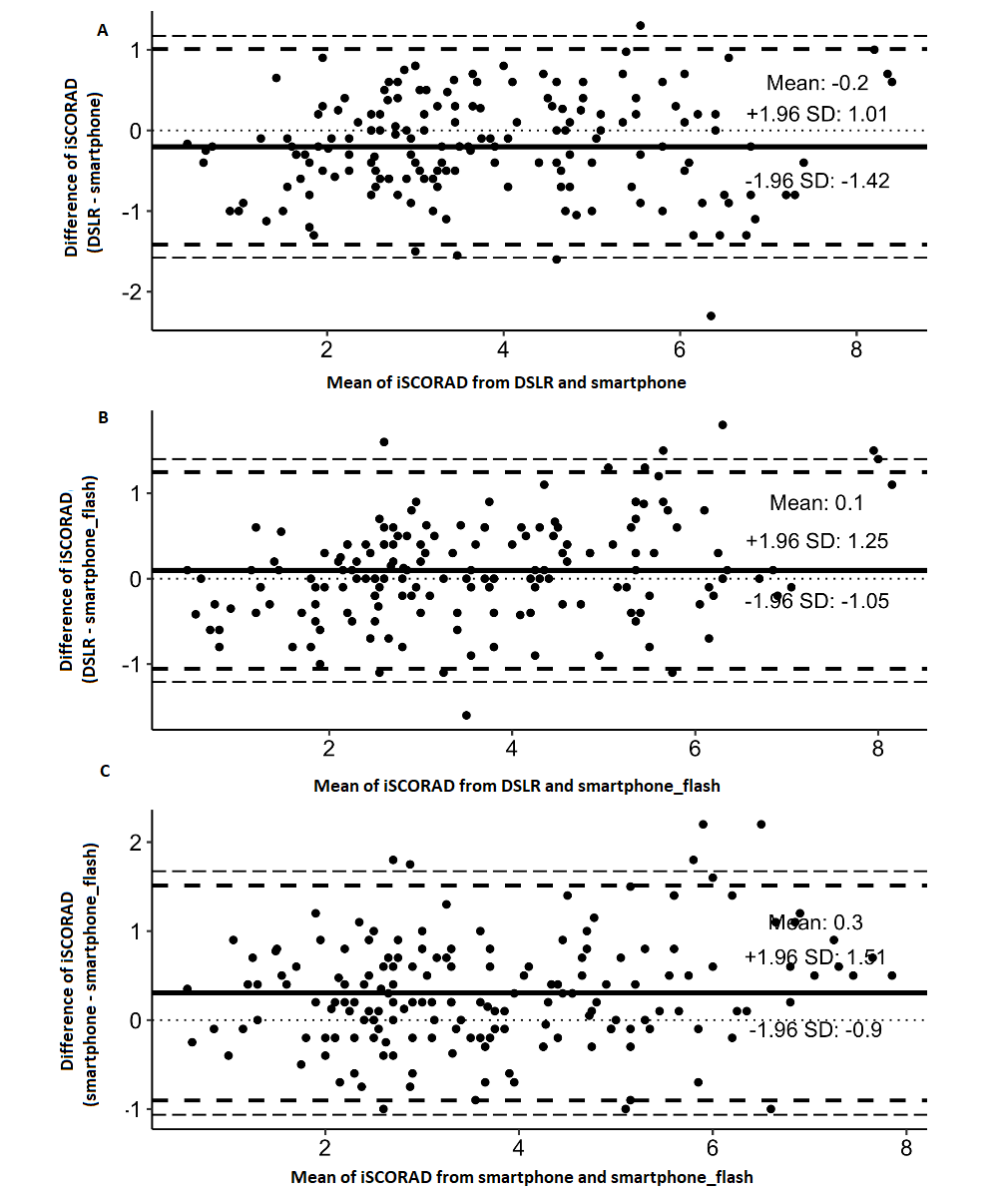
A Bland-Altman plot of the difference between the intensity items of Scoring AD (iSCORAD) remotely assessed based on (A) digital single-lens reflex camera (DSLR) camera and smartphone without flash, (B) DSLR camera and smartphone with flash, and (C) smartphone with and without flash. The solid line represents the mean difference, the broken line the 1.96 SD, and the dotted line represents zero.

The difference in mean scores for the Bland-Altman plot for the comparison between remote evaluations based on the different camera types was as follows: –0.2 for the DSLR camera and the smartphone without flash, 0.1 for the DSLR camera and the smartphone with flash, and 0.3 for the smartphone with and without flash (Figure 3).

## Discussion

### Principal Findings

In a setup where clinical assessments are conducted remotely, it is important to be certain that the assessments and clinical decisions made are similar to conventional clinical practice (in-person assessment). In this study, we showed that smartphone-based severity assessments are strongly correlated with in-person assessments. Further, photographs taken with a smartphone are similar to DSLR photographs in the assessment of AD severity using iSCORAD.

It has been demonstrated that the Psoriasis Area and Severity Index can be determined with moderate to good accuracy by dermatologists using standardized digital photos to assess the severity of psoriasis [13]. In patients with acne, Total Inflammatory Lesion Count was found to be the most reliable way to remotely track progress over time [14], whereas the Leeds technique and the Investigator’s Global Assessment designed to grade acne during in-person visits were not reliable in the assessment of digital photos of acne [14]. Further, a pilot study showed that a clinician viewing 3D photos could accurately measure and assess a diabetic foot ulcer remotely [15].

The assessment of AD severity relies on the assessment of clinical manifestations and subjective symptoms, as there is no specific and adequate serological or laboratory test to diagnose or monitor AD. A systematic review performed by Hill et al [16] found 62 different AD severity scales used in clinical trials, of which SCORAD was among the most commonly used. The level of agreement between different raters to give a consistent assessment of AD severity for the same patient has been investigated previously for SCORAD. Bozek et al [17] reported an ICC value of 0.66 for the intrarater reliability for objSCORAD with 10 trained dermatologists assessing 10 adult patients with AD. Zhao et al [18] also investigated in-person reliability for objSCORAD where 12 patients with AD were assessed by 5 trained dermatologists. In that study, an ICC of 0.498 (95% CI 0.234-0.785) and 0.446 (95% CI 0.037-0.730) for interrater and intrarater reliability, respectively, was reported. In another study with full-body photographs of 20 patients with AD of different skin colors assessed by 5 assessors showed that the interrater ICC for objSCORAD was –0.089 for highly pigmented patients, 0.588 for mildly pigmented patients, and 0.586 for nonpigmented patients [18].

In our study, there was a strong and significant correlation between in-person severity assessment and the 5 dermatologists’ remote assessments of photographs. Further, the degree of severity assessed remotely based on smartphone photographs was similar to those based on DSLR photographs. The widespread use and ownership of smartphones in the general public may suggest that, with the right training, patients may be able to use their own devices in clinical trials to photograph lesions without compromising the clinical evaluations.

### Strengths and Limitations

Our study has both important strengths and limitations that need to be addressed. It is a large study with 5 dermatologists rating hundreds of photographs remotely. The extent to which different camera types influence severity assessments based on photos has been investigated for the first time. In real-life settings, the photographs will often be taken by a smartphone and not a DSLR camera due to the ubiquity of smartphones in today’s society. In VCTs and teledermatology, the photographs will often be taken by the patients themselves and not by the clinician. Therefore, it is important to demonstrate that smartphones are valid tools to collect photographs that can be used to assess severity to the same degree as photographs taken by a DSLR camera. An important limitation is that the photographs used in this study are from a previously conducted study and therefore not collected for the purpose of this research. This explains why only iSCORAD was assessed remotely in our study, since information on disease extent, itch, and sleep quality was not available for remote assessments. Lastly, on average, the in-person intensity ratings were 1 point higher than the ones based on photographs. The trend appears to be linear, meaning that patients with greater severity are increasingly not being scored as “severe” in the remote assessments as they are in person. This could be due to lack of experience by the clinician rating the patients in person since the physician was not a trained dermatologist and the remote assessors were certified dermatologists with at least 5 years of experience in the field. Another explanation could be that the global overview the in-person physician has is lacking when remote assessments are done based on photographs. Future studies should therefore investigate interrater and intrarater reliability between in-person assessment and smartphone photographs taken by the patient at home to investigate the real-world scenario of future virtual trials and use in teledermatology.

### Conclusion

In conclusion, this large study based on 5 dermatologists’ assessments of hundreds of photographs showed that remote severity assessments are strongly associated with in-person assessments. We also found that smartphones are valid tools to collect photographs and can be used to assess AD severity to the same degree as photographs from a DSLR camera. Further, variation in how the dermatologists rated the iSCORAD based on the photographs was very low. Although this study clearly demonstrates the potential for remote severity assessment of AD, the validity and reliability of the photograph-based methodology should be investigated in a properly designed method-comparison study before implementation. 

## Abbreviations

AD: atopic dermatitis
ADES: Atopisk Dermatit Eksem Studie
DSLR: digital single-lens reflex camera
ICC: intraclass correlation coefficient
iSCORAD: SCORAD-intensity
objSCORAD: objective SCORAD
SCORAD: Scoring AD
VCT: virtual clinical trial
